# The Application of Ultrasound Pre-Treatment in Low-Temperature Synthesis of Zinc Oxide Nanorods

**DOI:** 10.3390/ma17204980

**Published:** 2024-10-11

**Authors:** Anna Drabczyk, Magda Ciężkowska, Katarzyna Kałahurska, Adam Zięba, Wojciech Bulowski, Katarzyna Bucka, Patryk Kasza, Krzysztof Zbroja, Grzegorz Putynkowski, Robert P. Socha

**Affiliations:** 1 CBRTP SA—Research and Development Center of Technology for Industry, 3A Ludwika Waryńskiego St., 00-645 Warsaw, Poland; mciezkowska14@gmail.com (M.C.); kkalahurska@gmail.com (K.K.); a.zieba@poczta.onet.pl (A.Z.); wojciech.bulowski@cbrtp.pl (W.B.); katarzyna.bucka@cbrtp.pl (K.B.); patryk.kasza@cbrtp.pl (P.K.); krzysztof.zbroja@cbrtp.pl (K.Z.); grzegorz.putynkowski@cbrtp.pl (G.P.); 2 Faculty of Non-Ferrous Metals, AGH University of Krakow, Al. Mickiewicza 30, 30-059 Kraków, Poland

**Keywords:** zinc oxide nanorods, nanorod crystallization, ultrasound treatment, particle agglomerate disintegration, ALD technique

## Abstract

Zinc oxide, due to its unique physicochemical properties, including dual piezoelectric and semiconductive ones, demonstrates a high application potential in various fields, with a particular focus on nanotechnology. Among ZnO nanoforms, nanorods are gaining particular interest. Due to their ability to efficiently transport charge carriers and photoelectric properties, they demonstrate significant potential in energy storage and conversion, as well as photovoltaics. They can be prepared via various methods; however, most of them require large energy inputs, long reaction times, or high-cost equipment. Hence, new methods of ZnO nanorod fabrication are currently being sought out. In this paper, an ultrasound-supported synthesis of ZnO nanorods with zinc acetate as a zinc precursor has been described. The fabrication of nanorods included the treatment of the precursor solution with ultrasounds, wherein various sonication times were employed to verify the impact of the sonication process on the effectiveness of ZnO nanorod synthesis and the sizes of the obtained nanostructures. The morphology of the obtained ZnO nanorods was imaged via a scanning electron microscope (SEM) analysis, while the particle size distribution within the precursor suspensions was determined by means of dynamic light scattering (DLS). Additionally, the dynamic viscosity of precursor suspensions was also verified. It was demonstrated that ultrasounds positively affect ZnO nanorod synthesis, yielding longer nanostructures through even reactant distribution. Longer nanorods were obtained as a result of short sonication (1–3 min), wherein prolonged treatment with ultrasounds (4–5 min) resulted in obtaining shorter nanorods. Importantly, the application of ultrasounds increased particle homogeneity within the precursor suspension by disintegrating particle agglomerates. Moreover, it was demonstrated that ultrasonic treatment reduces the dynamic viscosity of precursor suspension, facilitating faster particle diffusion and promoting a more uniform growth of longer ZnO nanorods. Hence, it can be concluded that ultrasounds constitute a promising solution in obtaining homogeneous ZnO nanorods, which is in line with the principles of green chemistry.

## 1. Introduction

Currently, nanotechnology plays an increasingly significant role in various industrial fields [1]. The sectors, where nanometer-sized materials serve as a crucial component, enhancing the functionality of the resulting products or providing unique properties, include the following, among others: medicine [2], agriculture [3], the food industry [4], petrochemical industry [5], biotechnology [6], and energy storage systems [7]. The high application potential of nanomaterials stems from their unique properties compared to macro-sized materials, which result from their larger surface area, quantum effects, and self-assembly [8]. Consequently, they exhibit outstanding electronic, optical, magnetic, mechanical, thermal, and catalytic properties [9,10].

Zinc oxide, due to its chemical and thermal stability, as well as high mechanical strength, holds promising potential for applications in nanotechnology and nanoscience [11]. Hence, among various types of nanomaterials, nano-sized forms of zinc oxide including nanoparticles, nanotubes, and nanorods (nanowires) have recently been widely investigated. For example, zinc oxide nanoparticles demonstrate a high electrochemical coupling coefficient, high chemical stability, and a broadened radiation absorption spectrum [12]. Importantly, ZnO nanoparticles also show antibacterial and antifungal activity [13,14]. The mentioned properties contribute to their wide use in fields such as medicine [15], dentistry [16], catalysis [17], agriculture [18], wastewater treatment [19], and the food industry [20]. Zinc oxide nanotubes have also recently attracted considerable interest from researchers. They have been investigated for catalysis [21], selective chlorides and fluorides adsorption [22], the detection of active substances [23], and targeted drug delivery [24].

Furthermore, ZnO demonstrates semiconductive and dual piezoelectric properties, a large direct bandgap, high breakdown voltage, and high exciton binding energy [11]. Hence, ZnO-based nanomaterials are being widely investigated in terms of their application for energetic, electronic, and optoelectronic industries as sensors or for energy conversion and energy storage uses. For example, Sedeh et al. [25] investigated ZnO nanotubes as modifying agents of polydimethylsiloxane-based surfaces considered as high-performance nanogenerators. Nanoadditives were deposited on polymer surfaces via a chemical bath deposition. It was demonstrated that such a modification positively affected both the open-circuit and short-circuit current of developed nanogenerators. In turn, numerous studies on ZnO nanorods have also been performed to develop supercapacitors [26,27,28], chemiresistive sensors [29], or materials for photovoltaic applications [30,31].

ZnO nanorods, due to their photoelectric properties and ability to efficiently transport charge carriers, have great potential in energy conversion and storage applications. In solar cells, they can enhance solar energy conversion efficiency. Their structure also supports energy storage, making them promising materials for batteries and other energy storage systems. There are several methods for synthesizing ZnO nanorods, including hydrothermal synthesis, in which the crystallization of ZnO nanorods occurs in an autoclave at high temperature (even up to 200 °C) and high pressure [32,33]. Other methods applied involve the sol–gel method [34,35], electrochemical deposition [36], chemical vapor deposition [37], thermal evaporation [38], and microwave-assisted synthesis [39]. While these methods are effective, they may involve significant energy inputs (high temperature and pressure conditions), high-cost equipment, long reaction times, or the need for further chemical or thermal treatment of the resulting nanorods. Hence, new and more sustainable synthesis methods are still being explored.

An interesting approach to the preparation of ZnO nanorods seems to be the application of ultrasound energy. So far, it has been utilized by some researchers. For example, Zhang et al. [40] employed ultrasounds (10 min), wherein the presented synthesis involved additional steps such as the centrifugation of the mixture after the sonication process. Moreover, the supernatant obtained was washed with an organic solvent (ethyl alcohol). A similar methodology involving the centrifugation of the mixture obtained as a result of sonication (5, 15, 30, and 60 min) and its further washing was also proposed by Zak et al. [41]. In other work, ultrasound energy was applied; however, the sonication process was performed for 2.0 h or 2.5 h, which is not economically favorable (high energy demand and time-consuming) [42].

In this paper, the low-temperature ultrasound-assisted chemical synthesis of zinc oxide nanorods has been proposed. Here, the short-term treatment of the precursor-containing solution with ultrasounds has been applied to obtain ZnO nanorods, wherein any additional steps like centrifugation have not been performed. The synthesis methodology has been designed to verify the effectiveness of ultrasound application, with the process being carried out for 1–5 min. As a reference, a synthesis without the sonication process has also been performed. Samples obtained as a result of all performed procedures have been imaged via scanning electron microscopy (SEM). Additionally, the particle size in the precursor suspensions has also been determined by means of dynamic light scattering (DLS method). Furthermore, the dynamic viscosity of precursor suspensions has also been evaluated.

## 2. Materials and Methods

### 2.1. Materials

Zinc nanorods were obtained using two main reagents, i.e., zinc acetate and sodium hydroxide. Sodium hydroxide was purchased from Stanlab Ltd. (Lublin, Poland). Zinc acetate dihydrate (ACS reagent, ≥98%) was bought in Chempur (Piekary Śląskie, Poland), whereas isopropanol (d = 0.785 g/mL) was purchased from Sigma Aldrich (Saint Louis, MO, USA). Trimethylaluminum (TMA) and diethylzinc (DEZ) used during the deposition process were purchased from Lanxess Organometallics GmbH, Berkgamen, Germany. All reagents were applied as received without further purification.

### 2.2. Preparation of the Substrate

**Figure 1:**
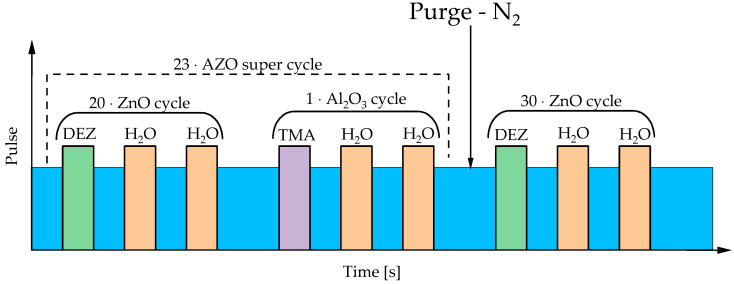
Scheme of the preparation of wafers acting as substrates during ZnO nanorod preparation.

For the growth of zinc nanorods, ultra-flat (0.18 mm thick) single-side polished Si wafers <100> (Alpha Nanotech Inc., Vancouver, Canada) were applied. Firstly, silicon wafers were exposed to argon plasma cleaning by means of the plasma cleaner Diener Tetra 30 (Diener electronic GmbH & Co KG, Plasma-Surface-Technology, Ebhausen, Germany). This process was performed for 2 min (power: 120 W, pressure: 0.3 mbar). The next step included the deposition of an aluminum-doped zinc oxide (AZO) layer containing additional zinc oxide crystallization nuclei on the silicon substrate (formation of AZO + ZnO-layered Si wafer). For this purpose, the atomic layer deposition (ALD) technique was employed. The process of deposition was conducted in a Beneq P400A (Beneq, Inc., Boulder, CO, USA) ALD system at 1 hPa of carrier gas pressure and 200 °C in the process chamber. TMA and DEZ were applied as sources of Al and Zn, while water was used as an oxygen precursor. The precursors were introduced to the chamber by means of a vapor draw. AZO deposition was achieved by supercycles of one pulse of DEZ followed by two pulses of water, whereby these twenty cycles were followed by one pulse of TMA followed by two pulses of water. This deposition procedure was repeated 23 times and was followed by ZnO deposition by 30 cycles of one pulse of DEZ followed by two pulses of water. Every precursor pulse was followed by the chamber purging for 2 s using nitrogen with 99.999% purity (PSA Nitrogen Gas Generator, Parker, Dukesway Gateshead, UK). The scheme of the process of AZO + ZnO-layered deposition on the Si wafer has been schematically presented in Figure 1.

Obtained wafers have been subsequently used as substrates during the synthesis of ZnO nanorods.

### 2.3. Synthesis of Zinc Oxide Nanorods

**Figure 2:**
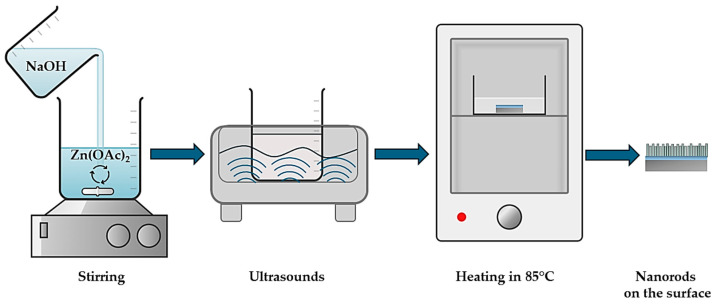
Scheme of ZnO nanorod preparation.

Firstly, a 1.0 M NaOH solution and a 0.25 M zinc acetate solution were prepared via dissolving adequate compounds in deionized water (25 °C, constant stirring 120 rpm). Next, 200 mL of zinc acetate solution was titrated with NaOH solution until pH = 7.50 was achieved. Then, the resulting mixture was subjected to the sonication (power 360 W) performed via the Digital Ultrasonic Cleaner (Walter Powersonic P 1100 D, Crest Ultrasonics Corp., Fremont, CA, USA) for 1–5 min, respectively. For comparison, the procedure without ultrasounds was also performed as a reference. Subsequently, the solution was poured into the Petri dish containing AZO + ZnO-layered Si wafer (fixed with two edges to the Petri dish via Kapton tape). The AZO + ZnO-layered Si wafers had additionally been cleaned with argon plasma (120 W, 0.3 mbar, 2 min) before their application as substrates for zinc nanorods. Finally, the Petri dishes with wafers treated with the solution obtained as a result of the titration were placed in a laboratory dryer (WAMED, Warsaw, Poland) at 85 °C for a specified time—i.e., 1.5 h or 2.0. After drying, wafers were removed and washed with deionized water and isopropanol. Every procedure was performed in triplicates. The procedure of nanorod synthesis has been schematically presented in Figure 2.

In Table 1, the reaction conditions of all samples have been compiled.

Next, all wafers with nanorods were subjected to SEM imaging. Importantly, performed investigations also involved determining the sizes of the particles contained in the reaction mixtures, as well as their dynamic viscosity.

### 2.4. Analysis of Samples’ Morphology via Scanning Electron Microscopy (SEM)

In order to verify the effectiveness of the conditions applied during the ZnO nanorod synthesis, obtained samples were analyzed via scanning electron microscopy. For this purpose, the Phenom Pharos (Thermo Fisher Scientific, Waltham, MA, USA) scanning electron microscope was applied. SEM imaging was conducted at 25 °C.

### 2.5. Analysis of the Particle Size Distribution within the Precursor Solutions via Dynamic Light Scattering (DLS)

An important aspect of performed works was to determine the influence of ultrasounds on the particle size distribution within the precursor solutions. For this purpose, precursor solutions after the sonication process (before annealing at 85 °C) were subjected to DLS analysis. The study was conducted by means of a Malvern Mastersizer 3000 analyzer (Malvern Panalytical Ltd., Nottingham, UK) equipped with a Hydro attachment enabling particle size measurement within the range of 0.01–3500 µm. The measurements were carried out at 25 °C.

### 2.6. Analysis of the Dynamic Viscosity of the Precursor Solutions

Conducted research also included determining the dynamic viscosity of precursor suspensions, as well as the impact of ultrasound treatment on this parameter and consequently, on the parameters of the nanorods obtained. The study was performed using Anton Paar Modular Compact Rheometer MCR 72 series (Anton Paar Poland Ltd., Warsaw, Poland). Measurements were conducted at 25 °C.

## 3. Results

**Figure 3:**
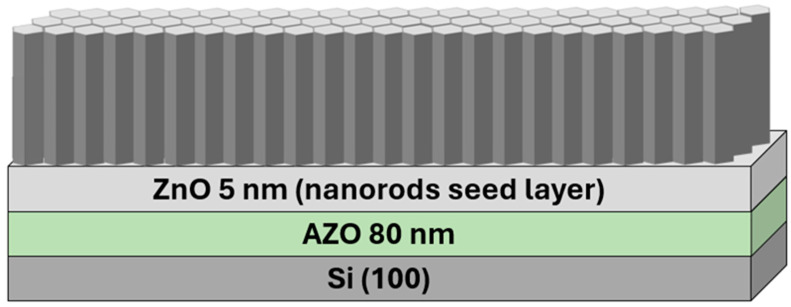
Scheme of manufactured system.

The obtained systems consisting of ZnO nanorods applied on the substate (AZO + ZnO-layered Si wafer) are schematically shown below in Figure 3.

Obtained ZnO nanorods were subsequently imaged via SEM, while precursor suspensions were analyzed in terms of verifying the size distribution of the precursor particles. Results of performed experiments are presented in Section 3.1 and Section 3.2.

### 3.1. SEM Imaging

**Figure 4:**
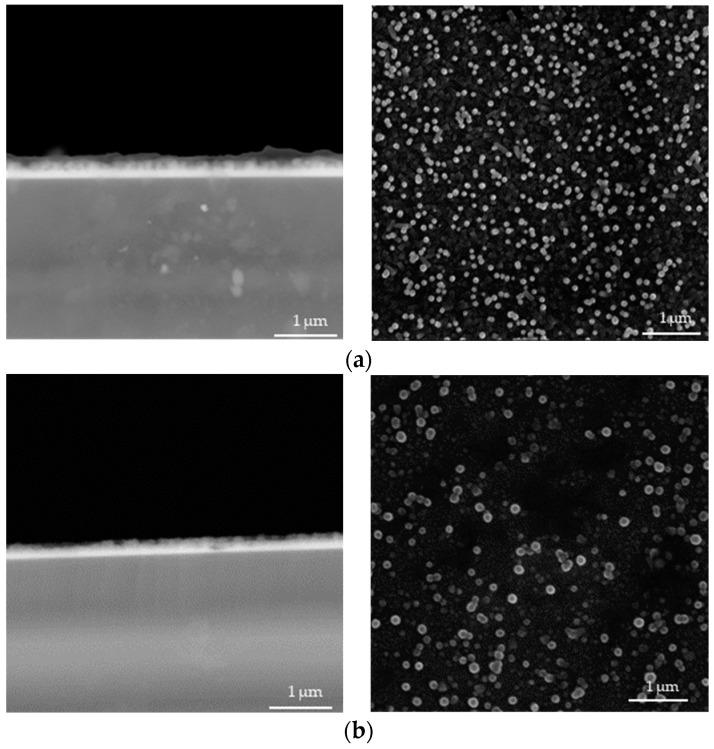
SEM images of nanorods obtained without ultrasounds and dried for 1.5 h (**a**) and 2.0 h (**b**) (**left**: cross-section image; **right**: surface image).

**Figure 5:**
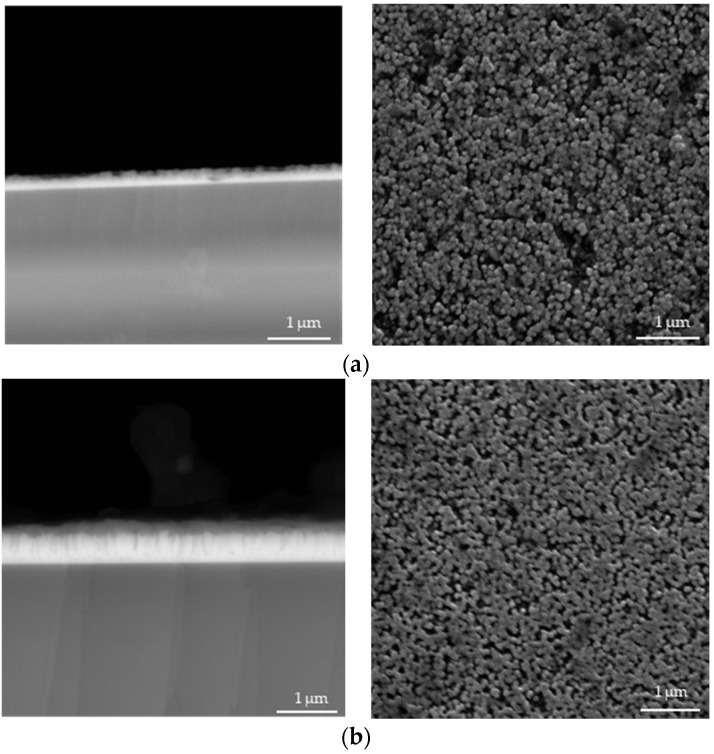
SEM images of nanorods obtained with 1 min of ultrasound and dried for 1.5 h (**a**) and 2.0 h (**b**) (**left**: cross-section image; **right**: surface image).

**Figure 6:**
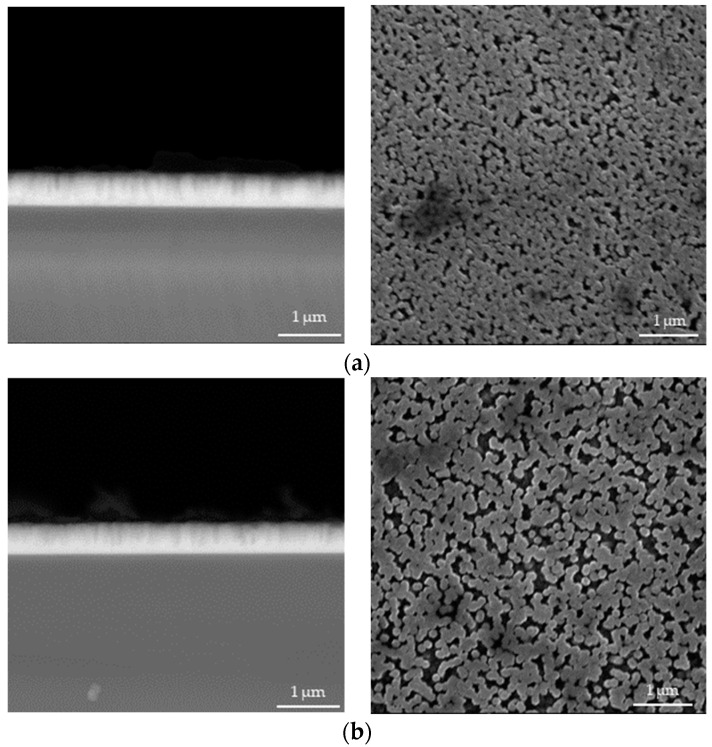
SEM images of nanorods obtained with 2 min of ultrasound and dried for 1.5 h (**a**) and 2.0 h (**b**) (**left**: cross-section image; **right**: surface image).

**Figure 7:**
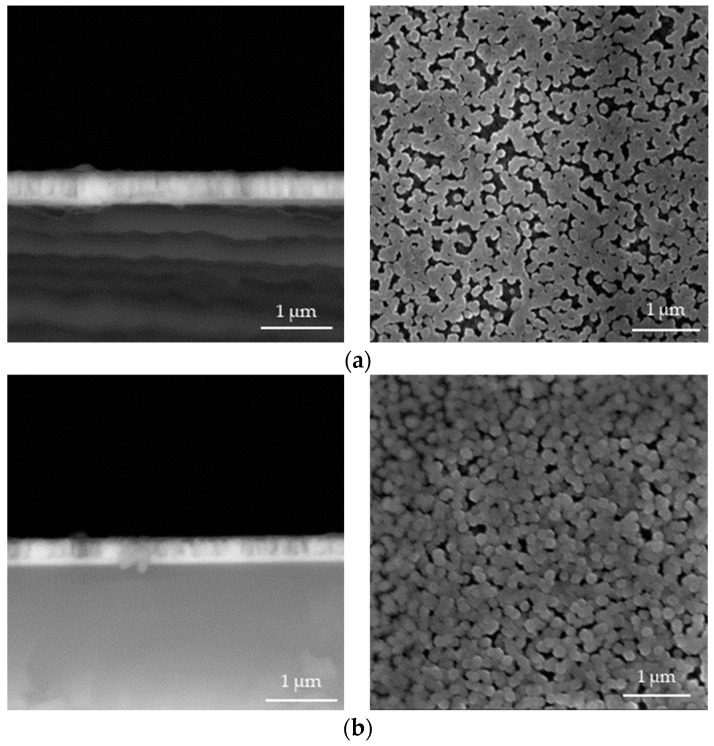
SEM images of nanorods obtained with 3 min of ultrasound and dried for 1.5 h (**a**) and 2.0 h (**b**) (**left**: cross-section image; **right**: surface image).

**Figure 8:**
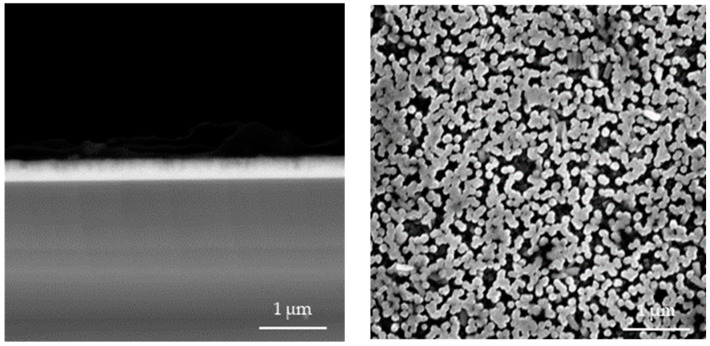
SEM images of nanorods obtained with 4 min of ultrasound and dried for 1.5 h (**left**: cross-section image; **right**: surface image).

**Figure 9:**
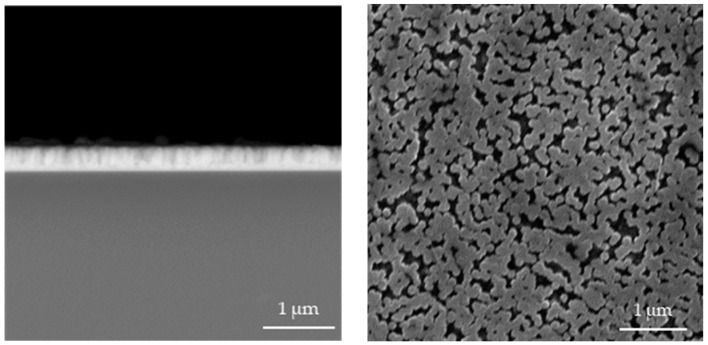
SEM images of nanorods obtained with 5 min of ultrasound and dried for 1.5 h (**left**: cross-section image; **right**: surface image).

In Figure 4, Figure 5, Figure 6, Figure 7, Figure 8 and Figure 9 below, SEM images of the obtained samples have been presented. In Table 2, parameters of the ZnO nanorods (their heights) have been compiled.

Based on the data presented above, it can be reported that ZnO nanorods obtained using ultrasounds demonstrated higher dimensions compared to the samples that were not subjected to sonication. In spite of that, there is no linear dependance between the applied sonication time and the height of obtained nanorods. Additionally, it can be observed that ZnO nanorods prepared via 2 h of heating at 85 °C have smaller dimensions, with a reduction of up to 21% for the sample sonicated for 1 min compared to these ones obtained using 1.5 h of heating. The simultaneous application of 2 h of heating and long-term sonication (i.e., 4 min and 5 min) did not enable the fabrication of ZnO nanorods.

### 3.2. Results of DLS Analysis

**Figure 10:**
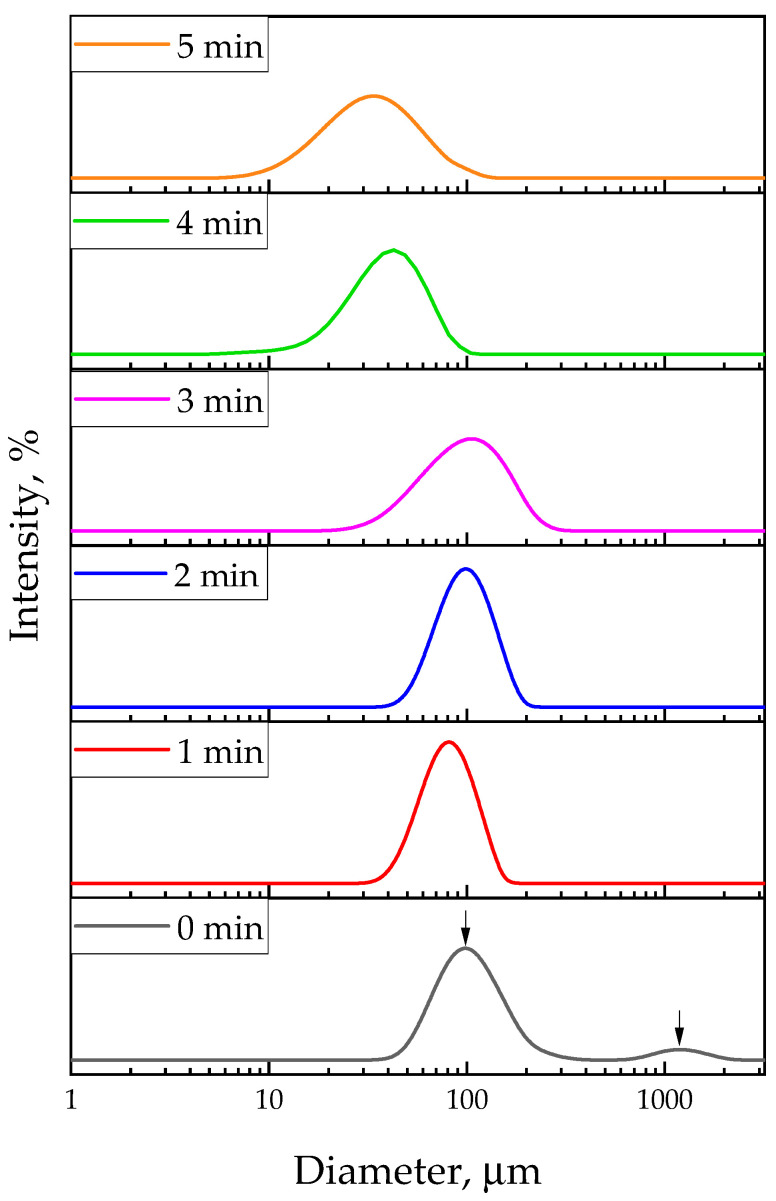
Particle size distributions of nanorod precursors in tested suspensions.

In Figure 10, the results of the DLS analysis are presented, while Table 3 compiles selected parameters describing particle size distribution in tested suspensions depending on the applied sonication time.

As it can be observed, the sonication process significantly affects the particle size distribution. Measured samples not subjected to the ultrasounds contained two particle fractions, while samples treated with ultrasounds showed higher homogeneity.

### 3.3. Results of Dynamic Viscosity Measurements

**Figure 11:**
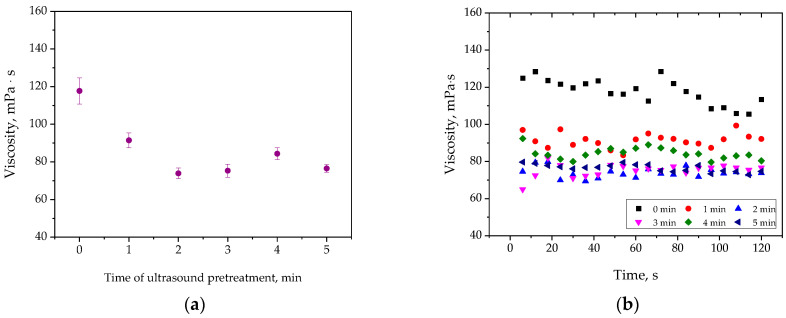
Values of dynamic viscosity of tested suspensions (graph shows average values for each sample and an error bar representing the standard deviation (SD)) (**a**) and dynamic viscosity change curve during the measurement (**b**).

Below—in Figure 11—the results of the viscosity tests of precursor suspensions are presented.

Based on the results obtained, it can be clearly stated that ultrasound pre-treatment resulted in the decrease in the dynamic viscosity of precursor suspensions. The dynamic viscosity of the suspension which was not subjected to ultrasounds was 117.69 ± 6.93 mPa · s, while the values of this parameter determined for samples after sonication was within the range of 73.93 ± 2.79 mPa · s–91.46 ± 2.91 mPa · s.

## 4. Discussion

The crystallization of ZnO nanorods involves the controlled growth of ZnO crystals in a preferential direction, usually along the hexagonal axis of the wurtzite. The process begins with the formation of crystal nuclei from a precursor solution, in this case, a NaOH-neutralized zinc acetate solution. In the presence of appropriate conditions, such as high temperature (which occurs during annealing), ZnO particles aggregate along preferential crystallographic directions, leading to the formation of elongated structures [43,44]. The crystallization of ZnO nanorods proceeds in several stages, i.e., the nucleation of crystals, their growth, and then aging. In addition, the crystals can combine to form larger agglomerates. The parameters used during the ongoing syntheses have a significant impact on the crystallization process [45,46].

Based on the performed experiments, it can be concluded that the use of ultrasound supports the formation of ZnO nanorods. The average height of nanorods obtained as a result of each of the procedures conducted with ultrasounds is greater than the dimensions determined for those obtained without ultrasounds. This is probably due to the fact that ultrasounds promote uniform distribution of the reactants in the reaction solution, which in turn promotes homogeneous crystal growth. A good dispersion of the reactants because of the sonication process has also been reported by Rayathulhan et al. [47]. It should be noted, however, that there is a non-linear relationship between the time of ultrasound application and the height of the nanostructures obtained. Subjecting the reaction mixture to ultrasound for 1–3 min leads to nanostructures of similar size, while in the case of sonication carried out for 4–5 min, a decrease in the height of ZnO nanorods to about 246.67 ± 8.17 nm–271.67 ± 47.08 nm can be observed (regardless of the heating time used).

Hence, it can be concluded that the crystallization process of ZnO nanorods is also significantly promoted by the action of ultrasound on the initial suspension before annealing. Ultrasound causes agglomerates to break up and the homogeneous dispersion of Zn^2+^ and OH- ions in the solution, which promotes the uniform formation of crystal nuclei. Thus, during the annealing phase, the growth process of nanorods is more controlled, and the nanorods form in a more homogeneous manner. Ultrasound also helps prevent the formation of large irregular agglomerates, which is crucial for obtaining high-quality nanostructures. If ultrasound is applied for too long, it can have a negative impact on the crystallization process of ZnO nanorods. Excessive sonication can lead to excessive breakdown of particles and crystal nuclei, resulting in very small irregular structures instead of well-formed nanorods. Prolonged sonication can also cause aggressive disintegration of already-forming crystals, resulting in disruption of the later stage of crystal growth, leading to heterogeneous or too-small structures [48,49].

The passage of high-frequency sound waves through liquid is accompanied by the phenomenon of cavitation, i.e., the formation and implosion of microbubbles [50]. These implosions cause local increases in temperature and pressure, which in turn promotes the formation of new nucleation centers. A shorter application time of ultrasound leads to an even distribution of energy in the reaction solution, which in turn promotes the uniform growth of nanorods. On the other hand, prolonging the sonication of the reaction mixture can intensify cavitation phenomenon in the solution, resulting in the formation of more nucleation centers favoring the formation of smaller, more numerous crystals (samples 4_1.5 and 5_1.5).

Importantly, in the case of most samples, ZnO nanorods obtained as a result of 1.5 h of heating demonstrated higher dimensions (by up to 25%) compared to nanostructures prepared as a result of 2.0 h of heating. Hence, it may be concluded that prolonged heating can have a negative impact on the structure of nanorods. As a result of long heat exposition, various changes within the structure of nanorods may occur, affecting their final dimensions. Additionally, these changes, in combination with a potential reduction in the surface defects of zinc oxide nanorods, can lead to their restructuring, thereby decreasing their dimensions [51,52].

Nevertheless, it should be noted that in the case of the conditions applied for samples 4_2.0 and 5_2.0, ZnO nanorods have not been obtained. Thus, it can be concluded that the simultaneous use of longer sonication times and 2 h of heating is not conducive to obtaining ZnO nanorods. The combination of a long time of ultrasounds and heating can destabilize the crystallization process, as well as cause the degradation of the growing crystals and the inhibition of their further growth.

Based on the DLS analysis, it can be concluded that the application of ultrasound affects the homogeneity of particles in the tested precursor suspensions. In Figure 10, showing the particle size distribution in the suspension which has not been subjected to the ultrasounds, two peaks can be observed, indicating the polydispersity of the tested sample. The first peak represents the predominant fraction of particles with a diameter of about 100 µm, while the second one indicates the presence of a small number of particles with a diameter of about 1200 µm. On the other hand, in the case of the results of the DLS analysis of samples after sonication, the presence of only one peak can be observed, which indicates greater particle homogeneity. Thus, it can be concluded that the application of ultrasounds increased the homogeneity of the analyzed samples. The Dx (90) parameter determined for the sample after 1 min of sonication (Table 3) is 115 μm, while the same parameter noted for the suspension not subjected to sonication is 200 μm. A significant reduction in the median particle size distribution (Dx (50)) from 104.0 μm to 79.8 μm was also observed. Therefore, it can be concluded that already after 1 min of sonication, particle agglomerates were disintegrated, resulting in a significant reduction in the size distribution of particles present in the tested suspension. After 2–3 min of ultrasound, a reduction in particle size was also observed compared to the suspension without the sonication process, with 90% of the particles having a diameter equal to or less than 141 μm (after 2 min) or 166 μm (after 3 min). Ultrasound-supported particle de-agglomeration resulting from the cavitation effect, among others (including nucleation, growth, and rapid collapse of microbubbles), was also discussed in the literature [53,54].

Analyzing the results of SEM imaging, it was observed that nanoparticles with the highest median height were obtained from suspensions subjected to sonication for 1–3 min. In the course of further analysis, it was also found that the application of sonication for longer time, i.e., 4 and 5 min, led to obtaining particles with a median size distribution of 39.3 μm (after 4 min) and 32.5 μm (after 5 min), respectively. This in turn led to nanoparticles with significantly smaller heights compared to those obtained from reaction suspensions treated with ultrasounds for 1–3 min.

SEM imaging was performed to show the morphology of the nanorods, which also enabled us to determine their dimensions, while the DLS technique allowed us to characterize the size distribution of the precursor particles (so ions, complexes, or agglomerates) in the solution before the annealing process. Although there is no direct correlation between the results from these two analyses, it can be concluded that the homogenization of the precursor size of the particles after sonication promotes a more controlled growth of the nanorods. Due to the uniform dispersion of particles in the suspension, the growth of crystals in the annealing phase is more controlled, resulting in greater uniformity and longer nanorods.

**Figure 12:**
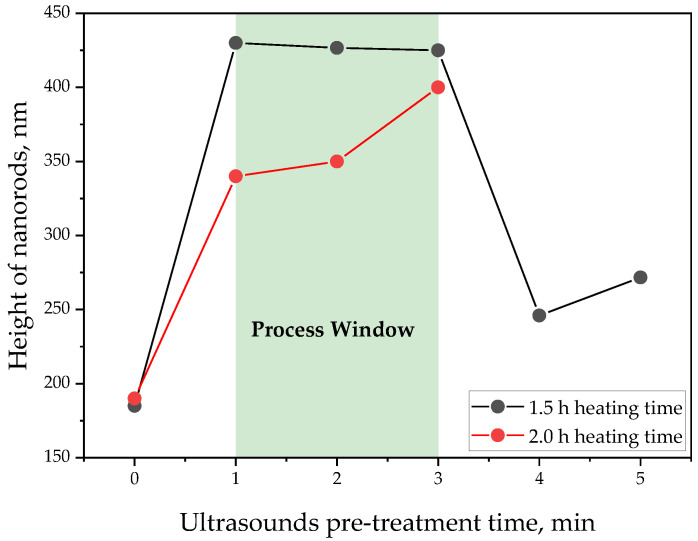
Process window of ZnO nanorod fabrication.

It was concluded that the most favorable sonication time leading to the fabrication of ZnO nanorods with the highest dimensions is within the range of 1–3 min. This has been presented schematically in Figure 12.

Observed dependance is probably due to the fact that smaller particles are more susceptible to the Gibs–Thomson effect, which describes the influence of the particles’ size on their solubility. The Gibbs–Thomson effect has a significant impact on the growth process of ZnO nanorods, especially with regard to the size and homogeneity of the crystals. This phenomenon describes the relationship between particle size and solubility—smaller particles have a higher surface energy, which makes them more susceptible to dissolution, as opposed to larger particles, which are more stable. Ultrasound can break down particles into very small sizes excessively, and smaller particles, due to the Gibbs–Thomson effect, demonstrate higher surface energy and higher solubility in a solution. As a result, smaller nuclei can dissolve faster instead of growing, which interferes with the crystallization process of nanorods. This phenomenon leads to the dominance of larger crystals at the expense of smaller ones, which can result in irregular and inhomogeneous structures. Thus, applying ultrasound for too long can disturb the balance between growth and the dissolution of nucleation seeds, negatively affecting the quality of nanorods [55].

Based on the performed measurements of dynamic viscosity, it was concluded that precursor suspensions subjected to the ultrasonic treatment demonstrated significantly lower dynamic viscosity compared to those not subjected to the sonication. As mentioned earlier, ultrasonic waves are accompanied by the phenomenon of cavitation, which involves the formation and implosion of microbubbles. These processes generate sudden local increases in pressure and temperature, leading to intense mixing of the medium subjected to the ultrasound. As a result of cavitation, agglomerates of particles present in the tested suspensions disintegrate. Consequently, particles resulting from the disintegration of agglomerates have a smaller contact surface area with the fluid compared to the original agglomerates, leading to reduced flow resistance and lower dynamic viscosity. In a less viscous medium, particles can diffuse more quickly through the solution to the growing ZnO crystals. As a result, a more homogeneous growth of nanorods is observed, which attains larger sizes.

## 5. Conclusions

- The use of ultrasound has a positive effect on the preparation of ZnO nanorods, as evidenced by the higher average heights of nanorods obtained with ultrasounds compared to those obtained without it. This is probably due to the fact that the sonication process promotes an even distribution of the reactants within the reaction mixture, which promotes uniform crystal growth.
- The duration of ultrasound affects the size of the resulting nanostructures. When the precursor suspension is sonicated for 1–3 min, longer nanostructures are obtained (340.00 ± 16.33 µm–430.00 ± 16.33 µm) compared to those obtained by subjecting the precursor suspension to ultrasounds for 4–5 min (246.67 ± 8.17 µm–271.67 ± 47.08 µm).
- The heating of the substrate with the applied reaction suspension for 1.5 h contributed in most cases to obtaining longer nanorods (by up to 26%) compared to the nanostructures obtained as a result of the process carried out with 2.0 h of heating.
- The use of ultrasound increases the homogeneity of the particles in the suspensions. The DLS analysis shows that sonication leads to the disintegration of particle agglomerates and thus to their greater dimensional homogeneity.
- Longer sonication times (4–5 min) lead to smaller nanoparticle heights, which may be due to the Gibbs–Thomson effect, where smaller particles show higher solubility and faster redistribution in the reaction mixture, which in turn leads to an increase in the number of nucleation centers. This increases the number of nucleation sites, which results in the formation of new crystals at various points while reducing the growth of already existing crystals. Hence, smaller particles contribute to the formation of shorter ZnO nanorods because of the simultaneous growth of a greater number of crystals.
- Ultrasonic treatment significantly reduces the dynamic viscosity of precursor suspensions, which in turn facilitates a faster diffusion of particles through the solution, promoting a more uniform growth of ZnO nanorods with larger dimensions.
- The developed methodology of ZnO nanorod preparation proceeded with a low energy input, a short reaction time, and without the need for the application of high-cost equipment; thus, it is promising and worthy of further investigations in line with current trends striving to obtain materials in line with green chemistry principles.

## Figures and Tables

**Table 1:** Reaction conditions of obtained samples.

No.	Time of Ultrasound Application, min	Heating Time, h	Sample Name
1.	0	1.5	0_1.5
2.	0	2.0	0_2.0
3.	1.0	1.5	1_1.5
4.	1.0	2.0	1_2.0
5.	2.0	1.5	2_1.5
6.	2.0	2.0	2_2.0
7.	3.0	1.5	3_1.5
8.	3.0	2.0	3_2.0
9.	4.0	1.5	4_1.5
10.	4.0	2.0	4_2.0
11.	5.0	1.5	5_1.5
12.	5.0	2.0	5_2.0

**Table 2:** Heights of nanorods obtained as a result of performed procedures.

Sample	Height of Nanorods, nm (Average Value ± SD)
0_1.5	185.0 ± 8.2
0_2.0	190.0 ± 8.2
1_1.5	430.0 ± 16.3
1_2.0	340.0 ± 16.3
2_1.5	426.7 ± 71.2
2_2.0	350.0 ± 16.3
3_1.5	425.0 ± 53.5
3_2.0	400.0 ± 16.3
4_1.5	246.7 ± 8.2
4_2.0	- *
5_1.5	271.7 ± 47.1
5_2.0	- *

* Applied conditions did not allow for the fabrication of ZnO nanorods.

**Table 3:** Values of Dx (10), Dx (50), and Dx (90) of analyzed samples.

Sonication Time, min	Dx (10) *, μm	Dx (50) *, μm	Dx (90) *, μm
0	65.9	104.0	200.0
1	53.8	79.8	115.0
2	65.3	97.2	141.0
3	50.5	96.7	166.0
4	21.2	39.3	64.3
5	16.0	32.5	62.8

* Dx (y) parameter indicates that y% of the particles have a diameter of this size or less.

## Data Availability

Data are contained within the article.
